# Establishing the cutoff value of near visual acuity for assessment of early presbyopia

**DOI:** 10.1007/s10384-024-01114-x

**Published:** 2024-08-31

**Authors:** Akiko Hanyuda, Miyuki Kubota, Shunsuke Kubota, Sachiko Masui, Kenya Yuki, Masahiko Ayaki, Kazuno Negishi

**Affiliations:** 1 Department of Ophthalmology, Keio University School of Medicine, 35 Shinanomachi, Shinjuku-ku, Tokyo, 160-8582 Japan; 2 Epidemiology and Prevention Group, Center for Public Health Sciences, National Cancer Center, Tokyo, Japan; 3 Department of Ophthalmology, Shonan Keiiku Hospital, Kanagawa, Japan; 4 Graduate School of Median and Governance, Keio University, Kanagawa, Japan; 5 Hazawa-Kubota Eye Clinic, Kanagawa, Japan

**Keywords:** Near visual acuity, Early presbyopia, Near activity visual questionnaire, Near-vision tasks

## Abstract

**Purpose:**

There is limited evidence to evaluate the numerical cutoff point for detecting early presbyopia. Thus, we aimed to establish a clinically relevant optimal cutoff value of near visual acuity for detecting early presbyopia.

**Study design:**

Prospective diagnostic accuracy study.

**Methods:**

We included consecutive individuals aged ≥ 20 years with a binocular-corrected distance visual acuity of ≥ 20/25 who did not undergo ophthalmic surgery between December 17, 2020 and December 19, 2021, at two healthcare facilities in Japan. Binocular distance-corrected near visual acuity at 40 cm, accommodative amplitude, awareness of presbyopia, and Near Activity Visual Questionnaire scores were examined. The optimal cutoff values of distance-corrected near visual acuity for diagnosing early presbyopia were evaluated using receiver operating characteristic plots.

**Results:**

Among 115 participants, 74 (64.3%) had presbyopia. The proportion of participants with no difficulty performing near-vision tasks decreased markedly when near visual acuity decreased to 20/20 (> 0.00 logMAR). A cutoff value of 0.00 logMAR for distance-corrected near visual acuity was optimal, showing high sensitivity of 56.76% and specificity of 92.68%, as opposed to the commonly used cutoff value of 0.40 logMAR (20/50; sensitivity, 9.46% and specificity, 100%) for diagnosing early presbyopia.

**Conclusion:**

Near visual acuity of 0.00 logMAR (20/20) could be the optimal cutoff value for diagnosing early presbyopia.

**Supplementary Information:**

The online version contains supplementary material available at 10.1007/s10384-024-01114-x.

## Introduction

Presbyopia, an age-related loss of accommodation, is the progressive inability to focus on near objects; it commonly begins to affect people in the mid-40s [1, 2]. With aging, protein alterations and the formation of molecules within the lens fiber cells may initiate the loss of lens elasticity, which is considered a primary cause of presbyopia [3, 4]. Uncorrected and under-corrected presbyopia cause a multitude of symptoms, leading to an enormous social and economic burden worldwide, since presbyopia develops and peaks during a patient’s prime working years [5]. With rapid global aging accelerating the decline in workforce and productivity [6], accurate diagnosis and proper management of presbyopia are of particular interest [7].

As presbyopia is one of the most common age-related ocular diseases [8], clearly defining this disease is becoming increasingly important for eye care providers to detect and treat it promptly and educate patients [9]. Wolffsohn et al. recently proposed this redefinition: “presbyopia occurs when the physiologically normal age-related reduction in the eye’s focusing range reaches a point, when optimally corrected for distance vision, that the clarity of vision at near is insufficient to satisfy an individual’s requirements” [2]. They also proposed an objective cutoff point for the severity of presbyopia based on the near add requirement, distance-corrected near visual acuity (DCNVA), and other refraction-related considerations [2]. Nonetheless, evidence on evaluating the numerical cutoff point for assessing early presbyopia remains limited.

Although the accommodative amplitude can objectively evaluate the presbyopic status in medical practice, near visual acuity (NVA) is likely the most accessible and commonly used method in presbyopia-related research [10]. Given that the expected accommodative amplitude ranges from − 4 D for mild-moderate presbyopia (requiring 1.0–2.0 D near add power) [11] and that a maximum amplitude of 2.6 D on average would be required when viewing a task at 40 cm [12], we aimed to explore the optimal cutoff value of NVA for the assessment of early presbyopia, characterized by a decreased accommodative amplitude of < 2.5 D in phakic eyes. We further examined whether presbyopia, defined using the optimal NVA cutoff value, reflects the patients’ presbyopic status in terms of self-awareness, decreased accommodative amplitude, or increased scores on the Near Activity Visual Questionnaire (NAVQ), the most appropriate questionnaire for assessing near vision in patients with presbyopia [13].

## Materials and methods

### Study population

We conducted a diagnostic accuracy study at the Shonan Keiiku Hospital (Kanagawa, Japan) and Keio University School of Medicine (Tokyo, Japan). We prospectively recruited consecutive healthy volunteers between December 17, 2020 and December 19, 2021. To maintain data integrity, the investigators at each institution met every 1–2 months to discuss the study protocol and examination procedures.

The inclusion criteria were: age ≥ 20 years and a binocular-corrected distance visual acuity (CDVA) of at least 20/25. The exclusion criteria were the presence of a cognitive disability, refusal to participate in the study; history of corneal or intraocular surgery, including ocular laser treatment and refractive or cataract surgery; moderate-severe cataract (≥ grade 2 nuclear cataract based on the WHO cataract grading system) [14], and severe dry eye disease (defined as positive symptoms of dry eye and a keratoconjunctival staining score of ≥ 3 points on the fluorescein staining test) because severe dry eye disease significantly affects NVA [15].

All participants provided written informed consent, and the study protocol was approved by the respective institutional review boards (approval numbers: 2020-K-8 for Shonan Keiiku Hospital; 20200157 for Keio University School of Medicine). The Standards for Reporting of Diagnostic Accuracy Studies guidelines were followed [16]. This study adhered to the tenets of the Declaration of Helsinki, and the protocol was registered with the University Hospital Medical Information Network Clinical Trial Registry (UMIN 000041819).

### Ophthalmic evaluations

Monocular CDVA, monocular DCNVA, binocular CDVA, and binocular DCNVA at 40 cm were measured according to the Japanese Industrial Standards [17]. We used the decimal visual acuity (VA) chart (distance VA: CV-7000, Tomey Co; near VA: TMI-V5040, T.M.I. Co.) and converted the decimal values to logMAR for statistical analyses. Accommodative amplitude and pupillary diameters were measured using an auto kerato-refractometer (ARK-1s; Nidek Co.). The participant’s head was positioned on a chin rest, focusing on a target that moved from distance to near vision. The built-in algorithm automatically calculated the accommodative amplitude by subtracting the initial objective refraction (i.e., the minimum refraction) from the maximum refraction achieved during a 30-s period. We measured the accommodative amplitude only once for each participant. In cases of measurement error, we reexamined the affected participants. Test strips containing fluorescein sodium (Fluores Ocular Examination Test Paper; Ayumi Pharmaceutical Co.) were used to evaluate tear break-up time (TBUT) and corneal staining scores. After applying two drops of saline solution to the test strip, we gently touched its edge to the inferior temporal lid margin. The participants were instructed to gently close their eyes and quickly open them. We measured the interval between the last complete blink and the appearance of the first dark black corneal spot with a stopwatch and regarded the average of three measurements as the TBUT. Corneal and conjunctival epithelial damage were evaluated using fluorescein vital staining and viewed through a blue-free filter; the staining scores were graded according to the van Bijsterveld grading system [18]. The ocular surface was divided into the temporal conjunctiva, cornea, and nasal conjunctiva, and the damage severity was scored in each section from 0 to 3 points (0: no damage to 3: damaged entirely), with the total score ranging from 0 to 9 points. Further, TBUT ≤ 5 s and corneal staining score ≥ 3 points were considered clinical dry eye signs [19, 20]. The examination room was maintained at 21–24 °C and 40–60% humidity. Trained ophthalmic technicians, who were unaware of the study design, conducted all the ophthalmic examinations except for the slit-lamp examination, which was performed by board-certified ophthalmologists.

### Assessment of subjective NVA and satisfaction

Subjective NVA and patient satisfaction were assessed using the 10-item NAVQ (Japanese version), validated to be modestly correlated with NVA (*r* = 0.32) and critical print size (*r* = 0.27), with an internal consistency of 0.945 and discriminant validity of 0.91 [13]. The subjective NVA was evaluated from 10 questionnaires, and each questionnaire was assigned a 4-point verbal descriptor scale: “no difficulty,” “a little difficulty,” “moderate difficulty,” and “extreme difficulty or stopped this activity due to vision.” The responses were converted to scores ranging from 0 to 100 on the Rasch scale, with higher scores indicating worse quality. Similarly, overall satisfaction was evaluated using a 5-point verbal descriptor scale ranging from “completely satisfied” to “completely dissatisfied.”

### Statistical analysis

Analyses were performed using SAS (version 9.4; SAS Institute); tests were two-sided, and significance was set at α = 0.05. We used data from the right eye in each participant, except while conducting binocular NVA tests. The mean values and standard deviations for continuous variables were tested using unpaired t-tests, and proportions for categorical variables were tested using the chi-squared test. Descriptive analyses were conducted to investigate the DCNVA relationship with accommodative amplitude and NAVQ scores. Accommodative amplitude was grouped into four sets (< 1.25, 1.25–2.5, 2.5–5.0, ≥ 5.0 D), and NAVQ scores were grouped into quartiles.

We primarily aimed to determine the optimal cutoff value of DCNVA for diagnosing early presbyopia. Given that a maximum amplitude of 2.6 D would be required on average for viewing a task at 40 cm [12], we defined early presbyopia as an accommodative amplitude of < 2.5 D in this study. We created receiver operating characteristic (ROC) curves to compare the ability of DCNVA to diagnose early presbyopia. The overall accuracy was summarized using areas under the ROC (AUROC) curves. Assuming a presbyopia prevalence of > 33%, the required sample size for an AUROC of 0.70 or 0.80 with a null hypothesis value (0.50) was < 72 and < 30, respectively. We assessed the sensitivity and specificity for DCNVA compared with those for early presbyopia at each cutoff value. The optimal cutoff value was determined using the Youden index (J = max [sensitivity + specificity–1]) [21].

Following that, we examined the distribution of accommodative amplitude and NAVQ according to the presbyopic status defined by the optimal NVA cutoff values. Stratified analyses by presbyopia awareness and the combined effects of presbyopia-related parameters were also conducted.

## Results

**Fig. 1:**
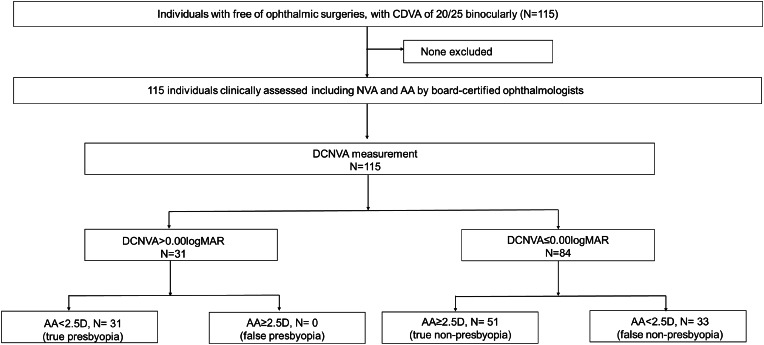
Study flowchart. AA, accommodative amplitude; DCNVA, distance-corrected near visual acuity at 40 cm

The study included 115 participants (73 men [63.5%]; mean age, 42.5 ± 10.8 years), 74 (64.3%) of whom had presbyopia (accommodative amplitude < 2.5 D) (Fig. 1). The participants’ baseline characteristics are shown in Table 1. Compared with the non-presbyopia group, participants in the presbyopia group were significantly older, more likely to be aware of presbyopia and had higher NAVQ scores (more presbyopic), lower DCNVA at 40 cm, lower accommodative amplitudes, and greater near pupillary diameters. The mean monocular and binocular DCNVA at 40 cm and accommodative amplitude were 0.12 ± 0.2 and − 0.04 ± 0.1 logMAR and 0.85 ± 0.6 D in the presbyopia group and − 0.12 ± 0.07 and − 0.14 ± 0.06 logMAR and 4.97 ± 1.5 D in the non-presbyopia group, respectively.

**Table 1:** Baseline characteristics and clinical features according to presbyopia (accommodative amplitude < 2.5 D)

Characteristics^a^	All patients (*n* = 115)	Presbyopia (*n* = 74)	Non-presbyopia (*n* = 41)	*p*-value^b^
Age (years)	42.5 (10.8)	47.8 (9.1)	32.9 (5.9)	**< 0.001**
Male sex, %	73 (63.5)	51 (68.9)	22 (53.7)	0.10
Awareness of presbyopia, %	65 (56.5)	47 (63.5)	3 (6.0)	**< 0.001**
Years of awareness of presbyopia	44.8 (4.8)	45.1 (4.6)	41.7 (7.6)	0.24
NAVQ score	23.3 (26.2)	29.1 (27.1)	12.9 (21.1)	**< 0.001**
**Monocular examination (right eye)**				
Subjective refraction, D	-3.55 (3.6)	-3.37 (3.7)	-3.89 (3.4)	0.46
Objective refraction, D	-3.81 (3.6)	-3.63 (3.7)	-4.12 (3.5)	0.49
CDVA, logMAR	-0.16 (0.05)	-0.16 (0.05)	-0.16 (0.04)	0.33
DCNVA at 40 cm, logMAR	0.04 (0.2)	0.12 (0.2)	-0.12 (0.07)	**< 0.001**
TBUT, s	6.16 (2.7)	6.34 (2.8)	5.83 (2.6)	0.34
Ocular surface staining, score	0.1 (0.3)	0.1 (0.3)	0.1 (0.3)	0.76
Accommodative amplitude, D	2.32 (2.2)	0.85 (0.6)	4.97 (1.5)	**< 0.001**
Distance pupillary diameter, mm	5.33 (0.8)	5.28 (0.7)	5.44 (0.9)	0.29
Near pupillary diameter, mm	4.36 (1.0)	4.57 (0.9)	3.99 (1.0)	**0.002**
**Binocular examination**				
CDVA, logMAR	-0.17 (0.03)	-0.17 (0.03)	-0.17 (0.02)	0.58
DCNVA at 40 cm, logMAR	-0.02 (0.2)	-0.04 (0.1)	-0.14 (0.06)	**< 0.001**

^a^Values are presented as means (standard deviations) for continuous variables and numbers (percentages) for categorical variables

^b^Unpaired t-tests and chi-squared tests were used, and p-values < 0.05 are marked in bold

CDVA, corrected distance visual acuity; DCNVA, distance-corrected near visual acuity; NAVQ, Near Activity Visual Questionnaire; SD, standard deviation; SE, spherical equivalent; TBUT, tear break-up time

**Fig. 2:**
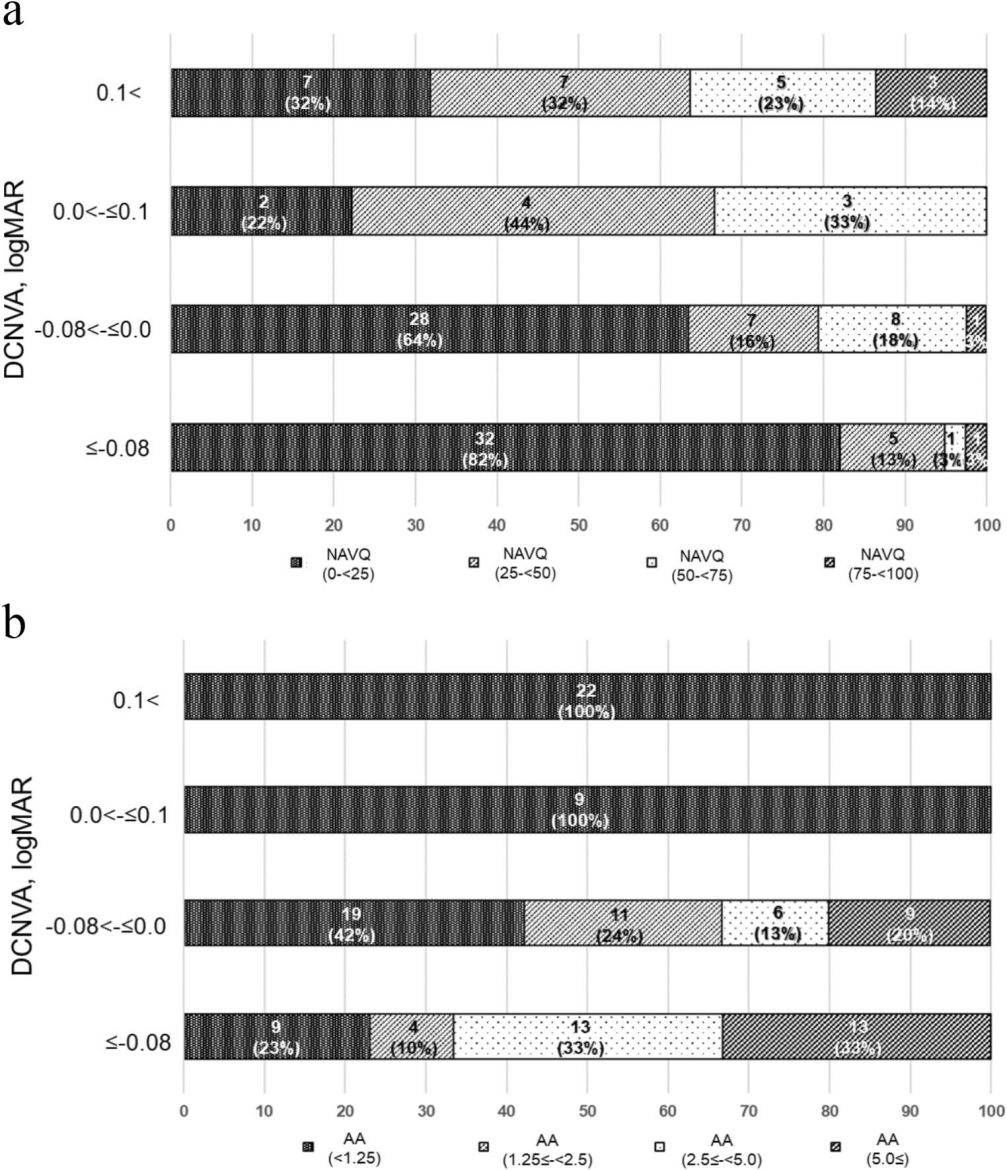
Distribution of (**a**) the NAVQ score and (**b**) AA stratified by DCNVA. (**a**) Over 80% of participants had no difficulty in performing near-vision tasks (the lowest quartile of the NAVQ score) in those with a DCNVA. The percentage of participants in the lowest quartile of the NAVQ score (0–25 points) decreases markedly in the group with a DCNVA of 0.0–0.1 and > 0.1 logMAR. (**b**) The accommodative amplitude decreases markedly when the DCNVA decreases to 0.0 logMAR. AA, accommodative amplitude; DCNVA, distance-corrected near visual acuity at 40 cm; NAVQ, near activity visual questionnaire; SD, standard deviation

The NAVQ scores and accommodative amplitude by DCNVA distributions are shown in Fig. 2. The lowest quartile percentage of NAVQ scores (0–25 points) markedly decreased in the groups with a DCNVA of 0.0–0.1 (Fig. 2a). In the groups with a DCNVA of ≤ -0.08 and − 0.08–0.0 logMAR, over 60% of individuals had low difficulty in performing near visual tasks (NAVQ score of 0–25 points), whereas this decreased to approximately 30% in those with a DCNVA of 0.0–0.1 and > 0.1 logMAR. Similarly, the accommodative amplitude was markedly reduced when DCNVA decreased to 20/20 (> 0.0 logMAR) (Fig. 2b).

**Fig. 3:**
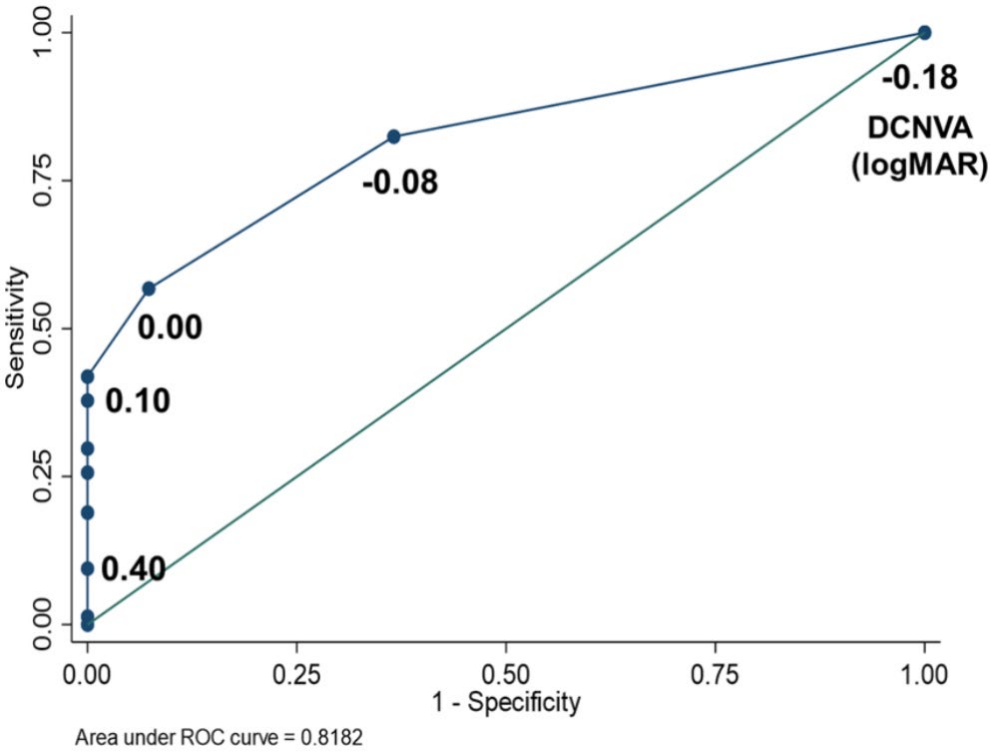
The area under the receiver operating characteristic (AUROC) for diagnosing presbyopia (accommodative amplitude < 2.5 D) using distance-corrected near visual acuity at 40 cm (DCNVA). The AUROC is 0.8182 (95% confidence interval [CI]: 0.748–0.888) with the optimal cutoff level of 0.00 logMAR for the DCNVA (sensitivity, 56.76%; specificity, 92.68%). The conventional cutoff values of 0.40 logMAR had a 9.46% sensitivity and 100% specificity

The ROC curves were evaluated to identify the optimal cutoff of DCNVA corresponding to early presbyopia (Fig. 3). The AUROC was 0.818 (95% confidence interval [CI], and 0.748–0.888) for DCNVA. Using the ROC plot, the optimal cutoff value for detecting early presbyopia was 0.00 logMAR for DCNVA (sensitivity: 56.76%; specificity: 92.68%). The commonly used cutoff value of 0.40 logMAR had low sensitivity, while the specificity was very high (sensitivity: 9.46%; specificity: 100%).

Table 2 summarizes the characteristics according to the presbyopic status defined by the optimal cutoff value of DCNVA (0.00 logMAR). On using the optimal cutoff value, 31 (27.0%) participants were presbyopic. Around 90% of participants in the presbyopia group were aware of their presbyopia, whereas only 26% in the non-presbyopia group presented with presbyopic symptoms (*p* < 0.001). The mean accommodative amplitude significantly decreased in the presbyopia group compared with the non-presbyopia group (0.48 ± 0.2 D vs. 3.00 ± 2.3 D, *p* < 0.001) (Table 2 and Online Resource 1). However, the NAVQ score was significantly higher in the presbyopia group than in the non-presbyopia group (presbyopia: 39.5 ± 24.4; non-presbyopia: 17.3 ± 24.3; *p* < 0.001) (Table 2 and Online Resource 2).

**Table 2:** Relationships of Presbyopia characterized by DCNVA with other presbyopia-related parameters

Characteristics^a^	DCNVA, logMAR		*p*-value^c^
	> 0.00 (presbyopia^b^)	≤ 0.00 (non-presbyopia)	
Patients, n	31 (27.0)	84 (73.0)	
Awareness of presbyopia, n	28 (90.3)	22 (26.2)	**< 0.001**
Accommodative amplitude, D	0.48 (0.2)	3.00 (2.3)	**< 0.001**
NAVQ score	39.5 (24.4)	17.3 (24.3)	**< 0.001**

^a^Values are expressed as means (standard deviations) for continuous variables and percentages for categorical variables

^b^Accommodative amplitude < 2.5 D

^c^Unpaired t-tests and chi-squared tests were used, and p-values < 0.05 are marked in bold

DCNVA, distance-corrected near visual acuity at 40 cm; NAVQ, Near Activity Visual Questionnaire; SD, standard deviation

To evaluate the combined effects of objective parameters and subjective symptoms related to presbyopia, we examined the relation between DCNVA and presbyopia-related parameters, stratified by the awareness of presbyopia (Online Resource 3). When we categorized participants into presbyopia/non-presbyopia groups using the DCNVA, the presbyopia group had a significantly decreased accommodative amplitude, compared with the non-presbyopia group, regardless of presbyopia awareness (*p* < 0.001).

## Discussion

Our results revealed the optimal cutoff value of DCNVA for the assessment of early presbyopia. The diagnostic performance of NVA was high (AUROC > 0.70) with an optimal cutoff value of 0.00 logMAR for DCNVA. When classifying presbyopia using this cutoff value, subjective symptoms (awareness of presbyopia, high NAVQ score) and objective parameters (decreased accommodation) related to presbyopia were significantly correlated with the presbyopia status. These findings are clinically beneficial because the commonly used cutoff values of 20/40 and 20/50 at 40 cm may be insufficient to meet patients’ functional demands in the real world [10]. Considering comprehensive data on subjective symptoms and objective findings, we propose that an NVA of 0.00 logMAR (20/20) can be a good and clinically relevant cutoff value for defining early presbyopia.

This study indicates that a cutoff value of 0.00 logMAR for DCNVA corresponds to the conventionally proposed presbyopic status (defined as an accommodative amplitude of < 2.5 D). Remarkably, this cutoff value of 20/20 is much higher than indicated in previous studies [10]. For instance, the World Health Organization defines uncorrected presbyopia as the inability to read either N6 or N8 binocularly (corresponding to 20/40 or 20/50) at 40 cm [10]. Conversely, our current findings are generally consistent with those of a previous survey suggesting that presbyopia was recognized in over 80% of individuals when NVA was decreased to 20/20 [22]. McDonald et al. recently used a DCNVA of 20/25 as the cutoff value for mild presbyopia and non-presbyopia [11]. In this study, a cutoff value of 0.00 logMAR correlated well with presbyopia subjective symptoms; presbyopia awareness was 90.3% in those with > 0.00 logMAR but dropped to 26.2% in those with ≤ 0.00 logMAR. Similarly, the lowest quartile distribution of NAVQ scores (representing no difficulty in near-vision tasks) significantly decreased from 64+ % in those with ≤ 0.00 logMAR to 22% in those with 0.00–0.10 logMAR. Our data further support that an NVA of at least 20/20 is required to perform near-vision tasks comfortably.

Near pupillary diameter was significantly greater in the presbyopic group compared with those with non-presbyopia, suggesting that it could be a potential predictor of presbyopia at an early stage. A close correlation between accommodative amplitude and changes in pupil size was reported among ophthalmologically healthy subjects aged 26–52 years [23]. Additionally, the positive relationship between accommodative amplitude and changes in pupillary diameter (the difference of near and distance pupillary diameters) remains consistent, regardless of age [24]. However, as measuring pupillary diameter requires advanced ophthalmic instruments and our primary goal was to explore a simple, accessible method for detecting early presbyopia, we focused on NVA as an alternative indicator of accommodative amplitude in this study. Future studies should investigate the utility of near pupillary diameter in detecting early stages of presbyopia.

The discrepancy between subjective symptoms and objective findings related to presbyopia is being increasingly recognized. These differences are likely in people with low-to-moderate myopia since they can focus on objects without needing accommodation effortlessly than those with emmetropia or hyperopia [25]. Other factors, including contrast sensitivity, the presence of a cataract, and/or stability of the ocular surface, can also result in such gaps, accounting for the complexity of managing presbyopia [2]. When we defined presbyopia using DCNVA, the presbyopia group had a significantly lower accommodative amplitude than the non-presbyopia group, regardless of presbyopia awareness. Our results suggest that 20/20 DCNVA can independently detect early presbyopia. In exploratory analyses, the ROC curve was stratified by the awareness of presbyopia and found that the optimal cutoff values were consistent for both DCNVA (0.00 logMAR), regardless of presbyopia awareness. Thus, it is likely that a DCNVA of less than the optimal cutoff value can be an independent determinant of presbyopia, irrespective of subjective symptoms.

Epidemiological studies are likely to differentiate presbyopia-based symptoms. Holden et al. divided presbyopia into “functional presbyopia” (needing an optical correction added to the presenting distance refractive correction to see either N6 or N8 optotype at 40 cm) and “objective presbyopia” (needing a significant optical correction [ ≥ + 1.00 D] added to the best distance optical correction to see either N6 or N8 optotype at 40 cm) [26]. Nonetheless, these definitions were generally empirically driven, and an NVA of 20/40 (or 20/50) might be too low for the endpoint of presbyopia correction, given our current lifestyle [22]. Based on our recent findings, we propose an accommodative amplitude < 2.5 D (or DCNVA less than 20/20 as a substitute for accommodative amplitude) as a cutoff value of early presbyopia. Although further studies are necessary to replicate our findings in different regions and among wider age groups, our definitions should be more practical, at least in developed countries.

A close connection between good health and the workforce has been proposed [26] and correcting near vision is expected to substantially improve global work productivity [5]. Although several barriers exist, including limited access to healthcare services, insufficient medical expenditure, and poor-quality glasses [27, 28], lack of disease awareness is a major cause of untreated presbyopia, especially among young individuals [29]. A recent report from rural China shows that one-third of participants (28.8%) did not receive near correction because their presbyopic condition was unrecognized [29]. This is consistent with the fact that approximately 20% of participants aged ≥ 45 years in developed countries were unaware of their presbyopia, despite having difficulty in near-vision tasks [22]. Therefore, making a clear threshold for intervention for presbyopia and education on the disease state are urgent public health concerns even in developed countries, considering that wearing glasses can successfully correct presbyopia, thereby retaining the workforce in the previous trials [30].

This study has certain limitations. First, selection bias may have affected our results because we recruited healthy participants with excellent access to affordable treatment. Also, this study included younger individuals than in previous near-vision-related studies [26]. Nonetheless, our results are less likely to be affected by various confounding factors such as the presence of cataracts, decreased contrast sensitivity, aberrations, increased light scattering, dry eye, or other vascular and inflammatory-related diseases commonly seen in older populations. To minimize being confounded by non-nuclear cataracts (i.e., waterclefts or retrodots) that may affect the CDVA, we performed sensitivity analyses among participants with CDVA ≥ 20/20 and confirmed the optimal cutoff value of 0.00 logMAR for DCNVA for detecting early presbyopia (69.44% sensitivity, 85.37% specificity, with AUROC curves of 0.839 [95% CI, 0.771–0.907]). Although further studies are warranted to achieve generalizability, the current findings from relatively young populations would provide clinically relevant treatments for addressing productivity loss due to uncorrected presbyopia. Second, only a few participants presented advanced presbyopia. Accordingly, a larger study with a different degree of accommodation is required to determine the finer categorizations of presbyopia. Third, we did not collect several important parameters relevant to presbyopia, such as the near point distance, deviation of the habitual refraction from the best subjective refraction, contrast sensitivity, or habitual reading distance. Nonetheless, the DCNVA and accommodative amplitude are most commonly used in the literature [10]. Finally, our study did not include pseudophakic eyes. Thus, our optimal NVA cutoff value is only suitable for diagnosing phakic presbyopia; additional studies are required for those who have undergone cataract surgery.

In conclusion, we propose a new cutoff value for the assessment of early presbyopia: DCNVA < 20/20. Considering the significant socioeconomic loss resulting from uncorrected presbyopia, a simple, accurate, and easily accessible method for assessing early presbyopia is urgently required. Although further studies are required to replicate our findings, the vision threshold reflecting the functional demands of daily life should be much higher than that commonly used in near-vision research (20/40) [10], at least in developed countries.

## Electronic supplementary material

Below is the link to the electronic supplementary material.

Supplementary Material 1
