# Supplementary material for: Establishing the cutoff value of near visual acuity for assessment of early presbyopia
Source: Jpn J Ophthalmol. 2024 Aug 31;68(6):709–16. doi: 10.1007/s10384-024-01114-x (PMC11607044; doi:10.1007/s10384-024-01114-x)
Supplement: Supplementary file 1 — Supplementary Material 1 [file 10384_2024_1114_MOESM1_ESM.pdf]

Supplementary Information

Establishing the Cutoff Value of Near Visual Acuity for Assessment of Early Presbyopia

Akiko Hanyuda,[1,2] Miyuki Kubota,[1,3,4] Shunsuke Kubota,[1,3,4,5] Sachiko Masui,[1] Kenya Yuki,[1] Masahiko Ayaki,[1] and Kazuno Negishi [1]

[1]Department of Ophthalmology, Keio University School of Medicine, Tokyo, Japan

[2]Epidemiology and Prevention Group, Center for Public Health Sciences, National Cancer Center, Tokyo, Japan

[3]Department of Ophthalmology, Shonan Keiiku Hospital, Kanagawa, Japan

[4]Graduate School of Median and Governance, Keio University, Kanagawa, Japan

[5]Hazawa-Kubota Eye Clinic, Kanagawa, Japan

Correspondence to:

Kazuno Negishi, MD, PhD

Department of Ophthalmology, Keio University School of Medicine

35 Shinanomachi, Shinjuku-ku, Tokyo 160-8582, Japan

Telephone: +81-3-3353-1211

E-mail: kazunonegishi@keio.jp

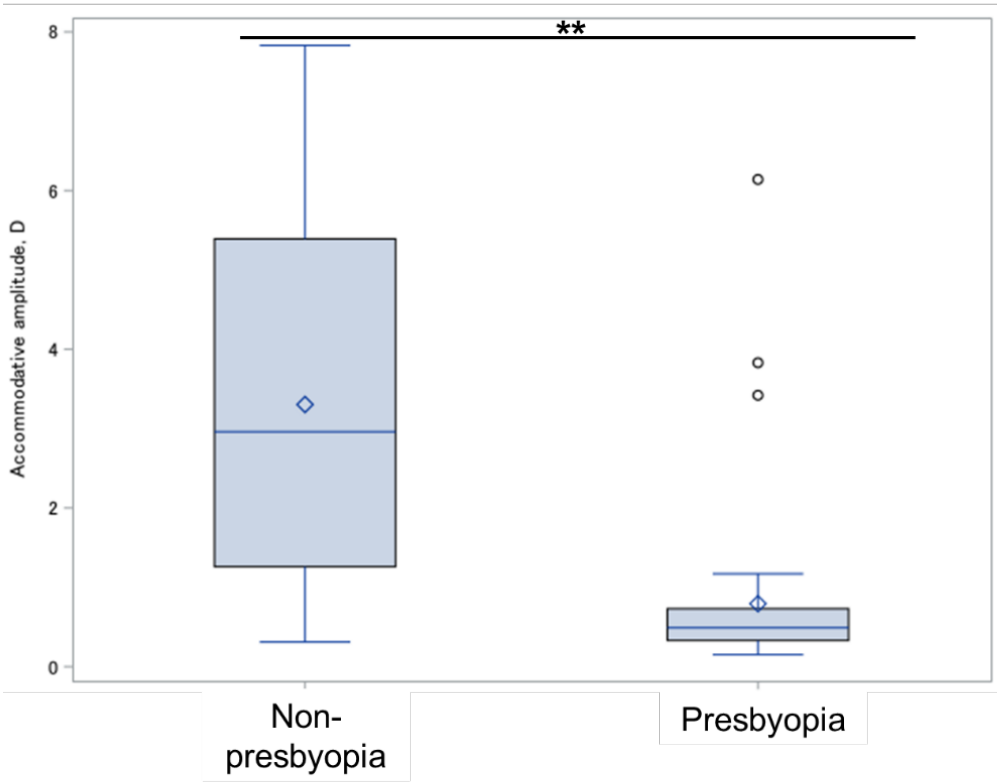

Online Resource 1. Distribution of accommodation amplitude according to presbyopia characterized by the DCNVA (>0.00 logMAR).

DCNVA, distance-corrected near visual acuity.

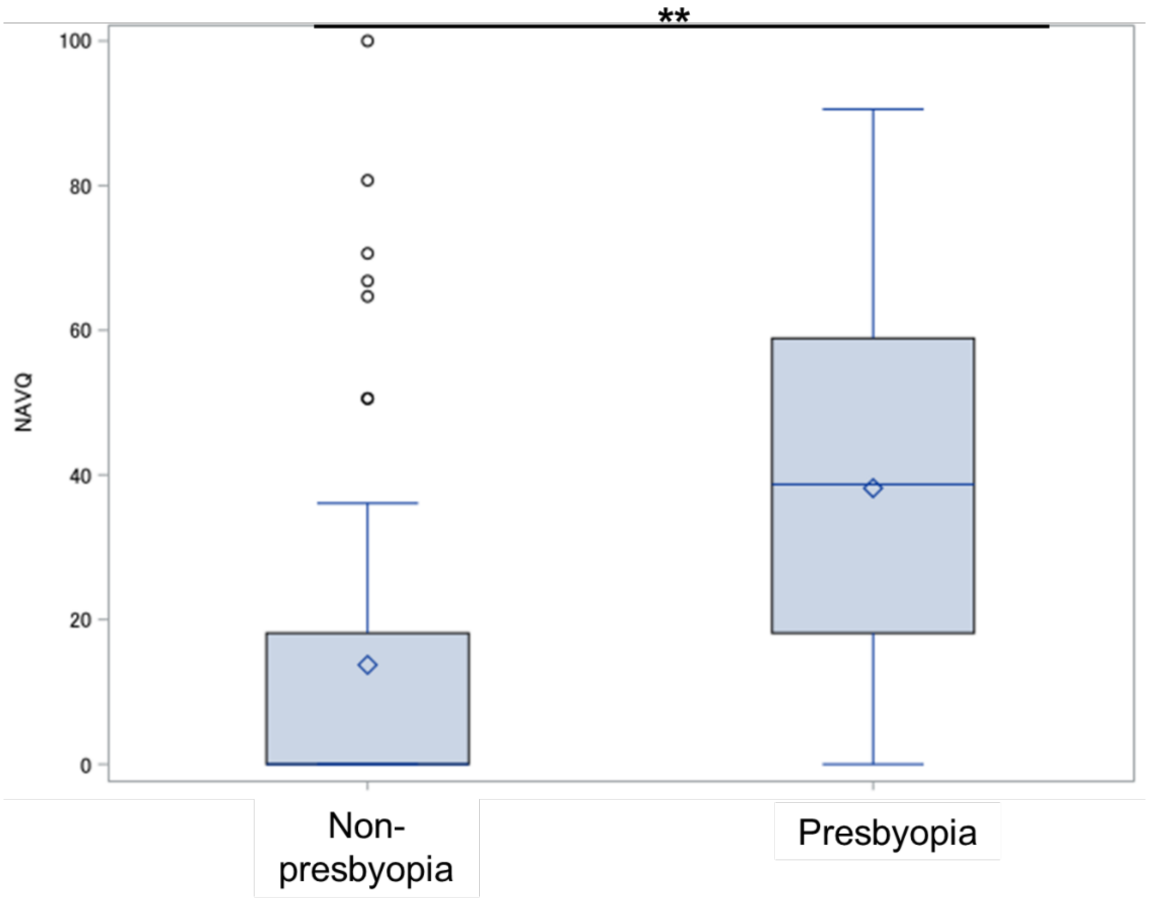

Online Resource 2. Distribution of NAVQ scores according to presbyopia characterized by the DCNVA (>0.00 logMAR).

DCNVA, distance-corrected near visual acuity; NAVQ, Near Activity Visual Questionnaire.

Online Resource 3. Relationships of presbyopia characterized by DCNVA with other presbyopia-related parameters, stratified by awareness of presbyopia

Characteristics[a]	**(-) Awareness of presbyopia**			**(+) Awareness of presbyopia**		
	**>0.00 (presbyopia)**	**≤0.00 (non-presbyopia)**	**p-value[b]**	**>0.00 (presbyopia)**	**≤0.00 (non-presbyopia)**	**p-value[b]**
Patients, n	3 (4.6)	62 (95.4)		28 (56.0)	22 (44.0)	
Accommodative amplitude, D	0.66 (0.2)	3.64 (2.2)	**<0.001**	0.46 (0.2)	1.20 (1.2)	**<0.001**
NAVQ score	16.5 (20.0)	10.4 (20.9)	0.63	42.0 (23.8)	36.7 (23.1)	0.43

[a]Values are presented as means (standard deviation) for continuous variables and percentages for categorical variables.
[b]Unpaired t-tests and chi-squared tests were used, and p-values <0.05 are marked in bold.

DCNVA, distance-corrected near visual acuity at 40 cm; NAVQ, Near Activity Visual Questionnaire; SD, standard deviation.
